# Association of adiponectin and socioeconomic status in African American men and women: the Jackson heart study

**DOI:** 10.1186/s12889-016-3167-x

**Published:** 2016-06-14

**Authors:** Sharon K. Davis, Ruihua Xu, Pia Riestra, Samson Y. Gebreab, Rumana J. Khan, Amadou Gaye, DeMarc Hickson, Mario Sims, Aurelian Bidulescu

**Affiliations:** Social Epidemiology Research Unit, National Human Genome Research Institute, National Institutes of Health, 10 Center Drive, Room 7 N320, MSC 1644, Bethesda, 20892 MD USA; University of Mississippi Medical Center, Jackson, USA; University of Indiana, School of Public Health, Bloomington, USA

**Keywords:** Biomarkers, Cardiovascular disease, Socioeconomic status, Adiponectin, African Americans

## Abstract

**Background:**

Recent emphasis has been placed on elucidating the biologic mechanism linking socioeconomic status (SES) to cardiovascular disease (CVD). Positive associations of inflammatory biomarkers provide evidence suggestive of a biologic pathway by which SES may predispose to CVD. African Americans have disproportionately lower SES and have a higher prevalence of CVD risk factors compared to most ethnic/racial groups. Adiponectin (an anti-inflammatory marker) is also lower. The objective of this study was to assess the association of adiponectin with SES among African American men and women using the Jackson Heart Study.

**Methods:**

Study sample included 4340 participants. Linear regression was performed separately by SES and stratified by sex. Annual household income and level of education was used as proxies for SES. Crude, age, health behavior and health status adjusted models were analyzed. The main outcome was log-transformed adiponectin.

**Results:**

Men in the lowest income group had significantly higher adiponectin than those in the highest income group in the fully adjusted model (ß/standard error [se], *p* value = .16/.08, *p* = .0008. Men with < high school level of education had significantly higher adiponectin in the crude and age adjusted models than those with ≥ college degree (.25/.05, *p* < .0001; .14/.05/ *p* = .005, respectively). Women with some college or vocational training in the crude and age adjusted models had lower adiponectin compared to women with ≥ college degree (−.09/.03, *p* = .004; −.06/.03, *p* = .04, respectively).

**Conclusion:**

Findings suggest a potential inverse biologic pathway between annual household income and adiponectin among African American men. There was no such finding among women. Findings suggest interventions should be targeted for higher SES African American men to improve adiponectin levels.

## Background

Research has consistently demonstrated an inverse association with socioeconomic status (SES) and cardiovascular disease (CVD) related mortality, morbidity and risk factors [1–4]. More recent emphasis has been placed on elucidating the biologic mechanism linking SES to CVD [5, 6]. Investigations show a significant inverse association between pro and anti-inflammatory biomarkers with SES which provide evidence suggestive of a biologic pathway by which SES may predispose to CVD [5–10]. These investigations suggest greater inflammation burden in those of lower SES. However, few studies have investigated the socioeconomic patterning of inflammatory biomarkers among African Americans. Those that include African Americans report adverse biomarker concentrations when compared to other racial/ethnic groups [9]. Research also shows lower SES African Americans have a higher prevalence of CVD risk factors [11]. Adiponectin is an anti-inflammatory biomarker that has increasingly been proposed as a risk factor for CVD related outcomes [12]. Adiponectin is a type of adipokine that is produced and secreted predominantly by adipocytes and lower levels has deleterious effects on the metabolic and vascular system which is inversely associated with type 2 diabetes and hypertension [13, 14]. Research demonstrates that African Americans have lower levels of adiponectin and a higher prevalence of obesity, hypertension and type 2 diabetes compared to other racial/ethnic groups in the United States [15, 16]. Lower SES is also disproportionately prevalent among African Americans [16]. We are aware of only three studies that compare adiponectin by SES [10, 17, 18]. To date, research has not been conducted in the United States that compares the association between adiponectin and SES among African American men and women or any other racial/ethnic group. Investigating the association between adiponectin and SES may provide important insights into the biological pathways linking SES with CVD and related risk factors in the higher risk population of African Americans. The objective of our study is to test the hypothesis that level of adiponectin may differ by SES among African American men and women and such association may be mediated by health status and health behavior.

## Methods

Data from the Jackson Heart Study (JHS) was used which is a single-site, prospective cohort of risk factors and causes of heart disease in adult African Americans. A probability sample of 5301 African Americans, aged 21–95, residing in three contiguous counties surrounding Jackson, Mississippi was recruited and examined at baseline from 2000 to 2004 by certified technicians according to standardized protocols [19, 20]. The present study includes cross-sectional data on 4340 participants who had complete data on all variables of interest. Those participants with missing values in the variables of interest were excluded. Baseline examination included blood pressure, anthropometry, survey of medical history, cardiovascular risk factors and collection of blood and urine for biological variables. Written consent was obtained from each participant before the collection of data. The study protocol was approved by the Institutional Review Boards of the National Institutes of Health and the participating JHS institutions-including the University of Mississippi Medical Center, Tougaloo College, and Jackson State University.

### Outcome variable

Adiponectin measurement was derived from venous blood samples drawn from each participant at baseline after more than 8 h of fasting. Vials of serum were stored at the JHS central repository in Minneapolis, MN, at −80 °C until assayed. Adiponectin concentration was measured in 2008–2012 as total plasma adiponectin by an ELISA system (R&D Systems; Minneapolis, MN. The inter-assay coefficient of variation was 8.8 %. No biological degrading has been described using stored specimens, indicating a high validity for measurement [21]. The distribution of plasma adiponectin values were positively skewed and were subsequently log-transformed for statistical analyses.

### Primary predictor variable

We used two measures of SES—self-reported pre-tax annual household income and educational level. Annual household income was divided into three categories (≤ $19,000, $20,000–$49,000, ≥ $50,000) and educational level was divided into four categories (<high school, high school or graduate equivalency diploma (GED), some college or vocational school, ≥ college graduate).

### Covariates

All covariates were collected at baseline and were chosen because they are associated with adiposity and hypertension related adiponectin [12]. Age was derived from date-of-birth. We further categorize covariates into health behavior and health status as mediator variables to determine if they would affect outcome. Health behavior variables include smoking status, physical activity, alcohol consumption status, and overweight based on body mass index (BMI) [16]. Smoking status was defined as current smokers and non-smokers. Physical activity was assessed with a physical activity survey instrument comprised of 4 domains (active living, work, home and garden, sport and exercise indexes). A total score was the sum of these domains with a maximum of 24 and a higher score indicates a higher level of physical activity. Alcohol consumption status was defined as “yes” if participant reported ever consuming alcohol and “no” for those reporting never consuming alcohol. Overweight was defined as BMI ≥25. Body mass index was derived from standing height and weight measured in lightweight clothing without shoes or constricting garments and calculated as weight in kilograms divided by height in meters squared (kg/m^2^). Cardiovascular disease health status variables include hypertension, type 2 diabetes, low-density lipoprotein (LDL), high-density lipoprotein (HDL), triglyceride, homoeostasis model assessment-insulin resistance (HOMA-IR), and C-reactive protein (CRP). Hypertension was based on a systolic blood pressure of ≥140 mmHg, diastolic blood pressure of ≥90 mmHg, or self-reported medication use for elevated blood pressure [14]. Blood pressure was measured using standard protocols with participant sitting quietly for 5 min measured at 1-min intervals. The average of two sitting blood pressure was used in the analysis. Type 2 diabetes was defined as fasting plasma glucose ≥126 mg/dL or self-reported use of insulin or oral hypoglycemic medications [13]. Fasting insulin, LDL, HDL and triglyceride were assessed using standard laboratory techniques. Insulin resistance status was estimated with HOMA-IR [22]. CRP was measured using immunoturbidimetric CRP-Latex assay from Kamiya Biomedical Company following manufacturer’s high-sensitivity protocol [23]. The inter-assay coefficients of variation on control samples repeated in each assay were 4.5 and 4.4 % at CRP concentrations of 0.45 and 1.56 mg/dL, respectively.

### Statistical analyses

All analyses were sex stratified because of the differential levels of adiponectin between men and women [24]. Descriptive analyses of the characteristics of men and women were performed using two sample *t*-test for continuous variables and chi-square for categorical variables. Characteristics of men and women stratified by annual household income and level of education were performed with one-way ANOVA for continuous variables and chi-square for categorical variables. Differential age adjusted mean levels of adiponectin for men and women by the three categories of income and four categories of education were graphed for descriptive purposes via one-way ANOVA. Adiponectin was based on sample mean.

Multiple linear regression analysis was utilized to test the association separately between two SES groups (i.e. annual household income, educational level) and log-transformed adiponectin with crude, age, health behavior and health status adjusted variables; ≥ $50,000 and ≥ college graduate were entered as referents for annual household income and level of education, respectively. Four separate models per SES group were utilized to test the effects on log-transformed adiponectin in a stepwise fashion. Model 1 was crude and included annual household income and educational level, while model 2 added age entered as a continuous variable. Model 3 added health behavior variables (BMI ≥25, smoking status, alcohol consumption status, physical activity score). Body mass index ≥25, smoking status, and alcohol consumption were entered as binary variables; physical activity was continuous. The fully adjusted model added health status variables (triglycerides, HDL, LDL, HOMA-IR, type 2 diabetes, hypertension and CRP). Triglycerides, HDL, LDL, HOMA-IR and CRP were entered as continuous variables; hypertension and type 2 diabetes were entered as binary variables. Tests of linear trends were also conducted to examine the association of SES sub-groups with levels of log-transformed adiponectin which were exponentiated (i.e. geometric means) after model adjustments based on general linear model. This was done to assess change in the magnitude of difference in adiponectin level between the highest and lowest SES sub-groups and to assess threshold effects between levels after adjustments as a measure of SES disparity and adiponectin level. A two-tailed level of significance was established as *p* ≤ .05. Analyses were conducted using SAS version 9.3 [25].

## Results

The sex-stratified characteristics of the study population are presented in Table 1. Women were significantly older than men (55 years of age versus 54, *p* = .002). A higher proportion of men had a higher household income of ≥ $50,000 compared to women (46 % versus 28 %, *p* < .0001). A higher proportion men were current smokers (17.7 % versus 11 %), consumed alcohol (60 % versus 40 %) and had a higher mean physical activity score (8.7 versus 8.2) when compared to women (*p* < .0001, respectively). Women on the other hand, had significantly higher mean BMI (33 kg/m^2^ versus 30 kg/m^2^, *p* < .0001). A higher proportion of women were also hypertensive (64 % versus 60 %), had type 2 diabetes (19 % versus 16 %) and had higher mean HDL cholesterol (55 mg/dL versus 46 mg/dL), HOMA-IR (3.7 versus 3.4), CRP (.60 mg/dL versus .37 mg/dL), and plasma adiponectin (6.0 ug/mL versus 4.1 ug/mL) when compared to men (*p* = .009, .003, <.0001, <.0001, <.0001, <.0001, respectively). Men had higher mean systolic and diastolic blood pressure (127.9 mmHg, 81.4 mmHg versus 126.2 mmHg, 77.2 mmHg), LDL cholesterol (128.7 mg/dL versus 125.5 mg/dL), and triglyceride (114.1 mg/dL versus 100.3 mg/dL) (*p* = .004, <.0001, .006, <.0001, respectively).

**Table 1:** Characteristics of study sample (*N* = 4340)

	Men	Women	*P*-value
	(*n* = 1604, 36.96 %)	(*n* = 2736, 63.04 %)	
Age (years), mean ± std.	53.86 ± 13.00	55.06 ± 12.63	.002
Socioeconomic Indicators			
Annual household income, %			
≤ $19,999	20.95	33.77	–
$20,000–$49,999	33.29	38.49	–
≥$50,000	45.76	27.74	<.0001
Educational level, %			
<high school	18.02	16.63	–
High school or GED	18.70	19.63	–
Some college or vocational	29.61	29.82	–
≥College graduate	33.67	33.92	.65
Health Behavior			
Current smoker,%	17.75	10.92	<.0001
Physical activity score, mean ± std.	8.71 ± 2.61	8.27 ± 2.57	<.0001
Alcohol consumption, %	60.24	39.80	<.0001
BMI(kg/m2), mean ± std.	29.84 ± 6.22	32.92 ± 7.63	<.0001
Health Status			
Hypertension,%	59.86	63.84	.009
Systolic blood pressure(mmHg), mean ± std.	127.9 ± 17.82	126.2 ± 18.42	.004
Diastolic blood pressure(mmHg), mean ± std.	81.45 ± 10.54	77.26 ± 10.12	<.0001
Type 2 diabetes,%	15.57	19.15	.003
LDL cholesterol (mg/dL), mean ± std.	128.7 ± 36.51	125.5 ± 36.21	.006
HDL cholesterol (mg/dL), mean ± std.	45.96 ± 12.45	55.19 ± 14.61	<.0001
Triglyceride level (mg/dL), mean ± std.	114.1 ± 90.22	100.3 ± 60.35	<.0001
HOMA-IR, mean ± std.	3.43 ± 2.39	3.78 ± 2.44	<.0001
C-reactive protein (mg/dL), mean ± std.	.37 ± 1.04	.60 ± 0.84	<.0001
Plasma adiponectin (ug/mL), mean ± std.	4.15 ± 3.36	6.09 ± 4.56	<.0001

*Abbreviations*: *std* standard deviation, *GED* graduate equivalency diploma, *BMI* body mass index, *LDL* low-density lipoprotein, *HDL* high-density lipoprotein, *HOMA-IR* homoeostasis model assessment-insulin resistance

There was a significant differential pattern of mean age and health behavior and health status according to annual income among men and women as revealed in Table 2. Men with an annual income of ≤ $19,000 were significantly older (57 years) than men in the other income categories (p < .0001). A higher percentage of men were also current smokers (27 %). Hypertension, systolic blood pressure, HDL cholesterol, and CRP were also significantly higher among this group of men (*p* = <.0002, .0012, .0006, .003, respectively). On the other hand, physical activity, alcohol consumption, BMI, diastolic blood pressure, and LDL cholesterol was significantly higher among men in the highest annual income category of ≥ $50,000 when compared to those in the other income groups (*p* = <.0001, .0003, .02, .0003, .0003, .001, respectively). Type 2 diabetes, and triglyceride were higher among those with an income of $20,000–$49,000 (*p* = <.0001, .03, respectively). Relatively the same pattern was observed among women. The exception was that those in the lowest income group of ≤ $19,000 had significantly higher BMI, type 2 diabetes, and triglyceride when compared to women in the other income groups (*p* = <.0001, <.0001, .003, respectively). Educational level with mean age and health behavior and health status patterned similarly among men and women as indicated in Table 3.

**Table 2:** Characteristics among men and women stratified by annual household income (*N* = 4340)

	Annual household income			
	≤$19,999	$20,000–$49,999	≥$50,000	*P*-value
Men (*n* = 1604)				
Age (years), mean ± std	57.56 ± 15.25	54.25 ± 13.09	51.87 ± 11.33	<.0001
Health Behavior				
Current smoker, %	27.16	18.64	12.77	<.0001
Physical activity score, mean ± std	7.51 ± 2.77	8.64 ± 2.54	9.30 ± 2.39	<.0001
Alcohol consumption, %	52.99	57.97	65.21	.0003
BMI (kg/m^2^), mean ± std	29.05 ± 6.97	29.93 ± 6.33	30.13 ± 5.75	.02
Health Status				
Hypertension, %	67.37	62.36	54.61	.0002
SBP (mmHg), mean ± std	129.87 ± 20.61	129.00 ± 17.72	126.13 ± 16.32	.0012
DBP(mmHg), mean ± std	79.42 ± 10.88	81.68 ± 10.89	82.20 ± 10.02	.0003
Type 2 diabetes, %	18.90	20.95	10.12	<.0001
LDL cholesterol (mg/dL), mean ± std	122.67 ± 37.74	128.51 ± 38.93	131.59 ± 33.83	.001
HDL cholesterol (mg/dL), mean ± std	48.32 ± 14.46	45.79 ± 12.27	45.05 ± 11.48	.0006
Triglyceride (mg/dl), mean ± std	107.90 ± 68.29	117.06 ± 75.47	114.69 ± 106.62	.03
HOMA-IR, mean ± std	3.16 ± 2.03	3.53 ± 3.02	3.49 ± 2.03	.10
CRP (mg/dL), mean ± std	0.52 ± 1.95	0.39 ± 0.67	0.29 ± 0.52	.003
Women (*n* = 2736)				
Age (years), mean ± std	58.67 ± 14.01	54.25 ± 11.91	51.80 ± 10.61	<.0001
Health Behavior				
Current smoker, %	15.25	10.21	6.62	<.0001
Physical activity score, mean ± std	7.59 ± 2.69	8.31 ± 2.51	9.04 ± 2.25	<.0001
Alcohol consumption, %	31.33	37.12	53.76	<.0001
BMI (kg/m^2^), mean ± std	33.49 ± 8.30	33.13 ± 7.58	31.92 ± 6.71	<.0001
Health Status				
Hypertension, %	72.19	64.11	53.26	<.0001
SBP (mmHg), mean ± std	129.88 ± 20.03	125.78 ± 17.82	122.46 ± 16.26	<.0001
DBP(mmHg)	76.83 ± 10.72	77.69 ± 10.08	77.19 ± 9.39	.16
Type 2 diabetes, %	25.88	17.37	13.47	<.0001
LDL cholesterol (mg/dL), mean ± std	126.47 ± 37.80	126.03 ± 35.89	123.69 ± 34.72	.26
HDL cholesterol (mg/dL), mean ± std	55.19 ± 15.19	55.32 ± 14.62	55.00 ± 13.93	.90
Triglyceride (mg/dl), mean ± std	105.42 ± 65.37	99.85 ± 58.84	95.12 ± 55.81	.003
HOMA-IR, mean ± std	3.87 ± 2.28	3.84 ± 2.60	3.63 ± 2.40	.14
CRP (mg/dL), mean ± std	0.64 ± 0.95	0.60 ± 0.79	0.53 ± 0.75	.02

*Abbreviations*: *std* standard deviation, *BMI* body mass index, *SBP* systolic blood pressure, *DBP* diastolic blood pressure, *LDL* low-density lipoprotein, *HDL* high-density lipoprotein, *HOMA-IR* homoeostasis model assessment-insulin resistance, *CRP* C-reactive protein

**Table 3:** Characteristics among men and women stratified by education (*N* = 4340)

	Education				
	< high school	High school or GED	Some college or vocational	≥ College graduate	*P*-value
Men (*n* = 1604)					
Age (years), mean ± std	65.05 ± 10.64	53.34 ± 12.73	48.92 ± 11.93	52.49 ± 11.67	<.0001
Health Behavior					
Current smoker,%	22.22	20.40	21.70	10.43	<.0001
Physical activity score, mean ± std	6.99 ± 2.46	8.38 ± 2.59	9.41 ± 2.52	9.18 ± 2.34	<.0001
Alcohol consumption, %	46.15	58.67	64.48	64.87	<.0001
BMI (kg/m^2^), mean ± std	28.57 ± 5.70	29.34 ± 5.58	30.60 ± 6.77	30.11 ± 6.21	<.0001
Health Status					
Hypertension, %	76.22	60.54	53.32	56.45	<.0001
SBP (mmHg), mean ± std	132.87 ± 20.89	128.47 ± 17.47	126.20 ± 16.96	126.26 ± 16.78	<.0001
DBP(mmHg), mean ± std	79.10 ± 10.76	81.50 ± 10.37	82.57 ± 11.01	81.69 ± 9.93	0.0002
Type 2 diabetes, %	24.03	16.89	13.33	12.26	<.0001
LDL cholesterol (mg/dL), mean ± std	126.54 ± 38.75	126.98 ± 36.27	129.01 ± 35.87	130.56 ± 36.05	0.4188
HDL cholesterol (mg/dL), mean ± std	48.34 ± 13.90	46.04 ± 11.86	45.51 ± 12.43	45.13 ± 11.91	0.0068
Triglyceride (mg/dl), mean ± std	109.24 ± 68.53	108.55 ± 62.68	123.60 ± 95.59	111.13 ± 105.56	0.0643
HOMA-IR, mean ± std	3.15 ± 1.89	3.42 ± 2.27	3.52 ± 2.00	3.51 ± 2.89	0.2609
CRP (mg/dL), mean ± std	0.59 ± 2.11	0.36 ± 0.62	0.32 ± 0.49	0.31 ± 0.60	0.0013
Women (*n* = 2736)					
Age (years), mean ± std	64.97 ± 10.11	57.11 ± 12.26	50.32 ± 11.97	53.20 ± 11.56	<.0001
Health Behavior					
Current smoker, %	13.00	11.63	12.95	7.69	0.0014
Physical activity score, mean ± std	6.69 ± 2.42	7.83 ± 2.60	8.71 ± 2.48	8.89 ± 2.33	<.0001
Alcohol consumption, %	20.13	33.90	44.35	48.86	<.0001
BMI (kg/m^2^), mean ± std	33.42 ± 7.75	33.06 ± 7.44	33.66 ± 8.21	31.93 ± 7.04	<.0001
Health Status					
Hypertension, %	82.52	69.81	57.76	56.55	<.0001
SBP (mmHg), mean ± std	132.34 ± 19.42	128.55 ± 20.03	124.12 ± 17.44	123.80 ± 16.92	<.0001
DBP(mmHg), mean ± std	75.21 ± 10.05	77.89 ± 10.27	78.00 ± 10.15	77.24 ± 9.92	<.0001
Type 2 diabetes, %	31.01	18.49	16.89	15.75	<.0001
LDL cholesterol (mg/dL), mean ± std	128.75 ± 37.36	127.76 ± 36.95	125.11 ± 37.74	123.06 ± 33.63	0.0259
HDL cholesterol (mg/dL), mean ± std	56.38 ± 16.02	55.70 ± 14.86	53.59 ± 13.28	55.74 ± 14.81	0.0084
Triglyceride (mg/dl), mean ± std	105.28 ± 54.41	98.73 ± 48.31	103.63 ± 67.07	96.02 ± 62.63	0.0196
HOMA-IR, mean ± std	4.08 ± 2.56	3.89 ± 2.50	3.84 ± 2.30	3.57 ± 2.48	0.0104
CRP (mg/dL), mean ± std	0.67 ± 1.04	0.58 ± 0.79	0.64 ± 0.85	0.53 ± 0.74	0.0104

*Abbreviations*: *std* standard deviation, *BMI* body mass index, *SBP* systolic blood pressure, *DBP* diastolic blood pressure, *LDL* low-density lipoprotein, *HDL* high-density lipoprotein, *HOMA-IR* homoeostasis model assessment-insulin resistance, *CRP* C-reactive protein

**Fig. 1:**
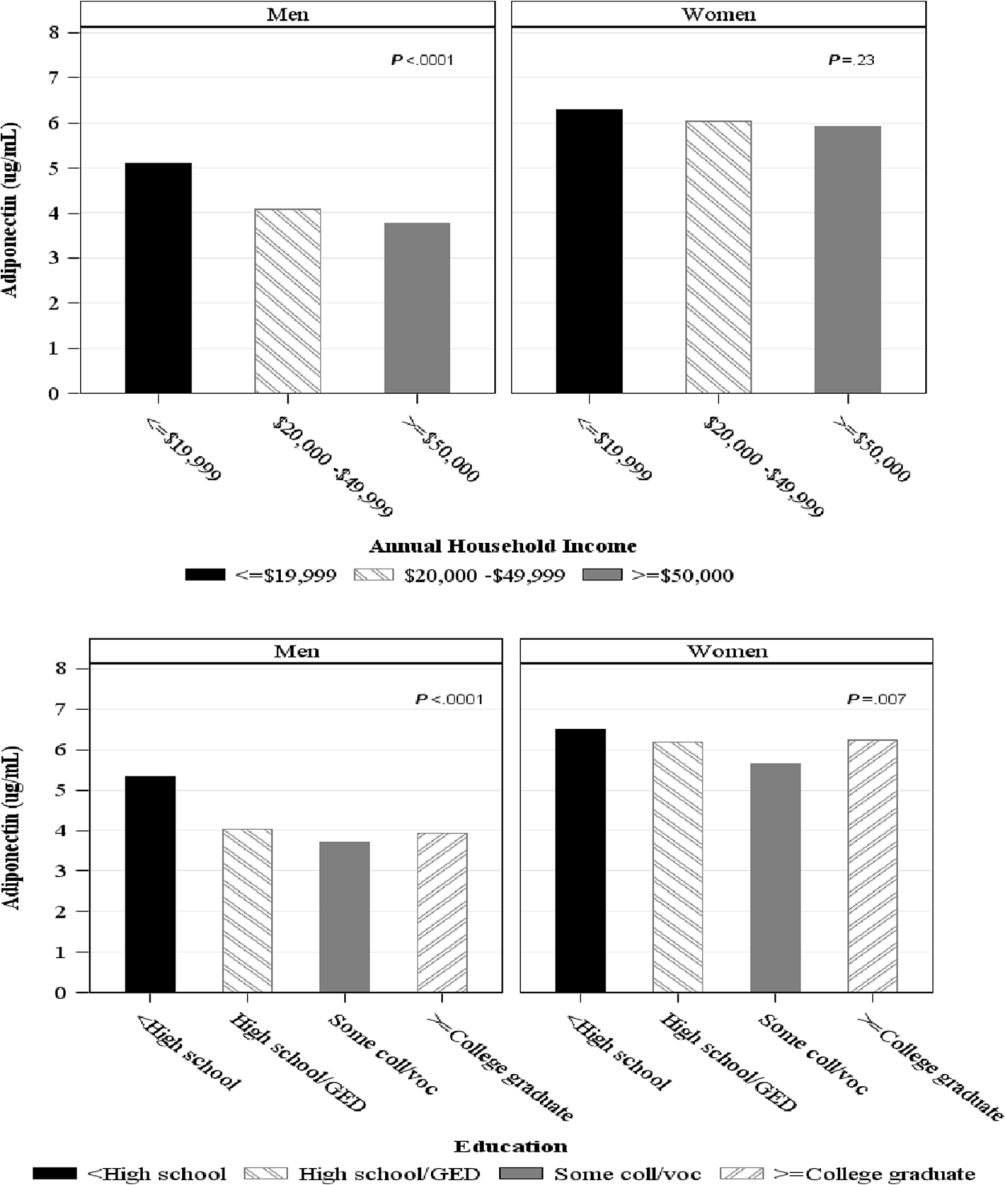
Mean age adjusted level of adiponectin for men and women by annual household income and level of education. *P* value represents differences in the level of adiponectin for income and education categories based on one-way ANOVA

Figure 1 illustrates mean age adjusted adiponectin was significantly different among men by annual household income with those in the lowest category having higher adiponectin (*p* < .0001). There was no difference among women. Mean adiponectin was also significantly different among men and women based on level of education with those with < high school level of education having higher adiponectin (*p* < .0001, .007, respectively).

Table 4 reveals men with a lower annual income of ≤ $19,999 had significantly higher plasma adiponectin when compared to men with a higher income of ≥ $50,000. This was evident in the crude model (β (se) = .26 (.04); *p* < .0001) and persisted in the fully adjusted model (β (se) = .16 (.05); *p* = .0008). The reduction in model coefficients ranged from 19 to 38 %. Those with a medium annual income of $20,000–$49,000 had marginally higher plasma adiponectin than men with a higher annual income of ≥ $50,000. Men with < high school level of education had significantly higher adiponectin in the crude model (β (se).25 (.05); *p* < .0001) and remained significant when adjusted for age (β (se) .14 (.05); *p* = .005), but significance disappeared after adjustment for health behavior and health status; the coefficients between both models were reduced by 44 %. There was no association among men with high school or GED level of education or among those with some college or vocational training.

**Table 4:** Regression coefficients with standard error of log transformed adiponectin by socioeconomic status for men and women (*N* = 4340)

Adiponectin											
Men (*n* = 1604)											
	^a^Model 1		^b^Model 2			^c^Model 3			^d^Model 4		
Socioeconomic status	β (se)	*P*-value	β (se)	*P*-value	% Red	β (se)	*P*-value	% Red	β (se)	*P*-value	% Red
Income											
≤ $19,999	.26(.04)	<.0001	.21(.04)	<.0001	19 %	.15(.05)	.0012	42 %	.16(.05)	.0008	38 %
$20,000 - $49,999	.07(0.04)	.056	.05(.04)	.17		.03(.04)	.43		.04(.04)	.25	
≥$50,000 (referent)	–										
Education											
<high school	.25(.05)	<.0001	.14(.05)	.005	44 %	.10(.05)	.06		.08(.06)	.16	
High school or GED	.05(.05)	.29	.04(.05)	.36		.01(.05)	.87		.03(.05)	.55	
Some college or vocational	–.009(.04)	.83	.02(.04)	.60		.01(.04)	.73		.001(004)	.96	
≥College graduate (referent)	–										
Women (*n* = 2736)											
Income											
≤ $19,999	.02(.03)	.53	−.04(.03)	.20		−.03(.03)	.38		−.01(.03)	.73	
$20,000–$49,999	0.01(0.03)	.71	−.01(.03)	.73		.008(.03)	.79		−.0002(.03)	.99	
≥$50,000 (referent)	–										
Education											
<high school	.02(.04)	.52	−.08(.04)	.03		−.05(.04)	.19		−005(.04)	.19	
High school or GED	−.02(.03)	.60	−.05(.03)	.12		−.03(.04)	.44		−.03(.03)	.35	
Some college or vocational	−.09(.03)	.004	−.06(.03)	.04	33 %	−.03(.03)	.31		.01(.03)	0.75	
≥College graduate (reference)	–										

*Abbreviations*: *SE* standard error, *Red* Reduction

Percentage reduction in regression coefficients from Model 1 computed by (regression_Model 1_-regression coefficient _Models 2, 3, 4_)/(regression coefficient_Model 1_)

^a^Model 1, unadjusted

^b^Model 2, adjusted for age

^c^Model 3, adjusted for age and health behavior (overweight, smoking status, alcohol consumption status, physical activity)

^d^Model 4, adjusted for age, health behavior and health status (HDL, LDL, triglycerides, hypertension status, type 2 diabetes status, HOMA-IR, CRP)

There was no significant association between annual household income and plasma adiponectin observed among women in any of the models. In the crude model, women with some college or vocational training had significantly lower levels of adiponectin than women with ≥ college level of education (β (se) .09 (03), *p* = .004). When adjusted for age, women with < high school level of education and those with some college or vocational training had significantly lower plasma adiponectin than women with ≥ college level (β (se)−.08 (.04), *p* = .03,−.06 (.03), *p* = .04, respectively). A 33 % reduction in coefficients between crude and age adjusted models was observed among those with some college or vocational training. There was no association in the models adjusted for health behavior or health status.

Table 5 presents the trends of log-transformed exponentiated level of adiponectin for income and level of education for each sub-group compared with the highest sub-group. The results show that men with an annual household income of ≤ $19,999 and $20,000–$49,000 had significantly higher mean adiponectin than those with an income of ≥ $50,000 (3.91 and 3.25 versus 3.02, *p* < .0001) in the crude unadjusted model. This pattern persisted in each of the subsequently adjusted models. There were no differences observed among women by level of income in the crude, age, health behavior, and health status adjusted models. In the crude model, men who had an education level of < high school and those with high school or GED, had higher levels of mean adiponectin than men with ≥ college level of education (3.98, 3.26 versus 3.10, *p* < .0001). A similar pattern was observed in the age-adjusted model (*p* = .006). But differences by education level were attenuated in the models adjusted for health behavior and health status. Women with an education of < high school, high school or GED, and those with some college or vocational training had lower levels of adiponectin than women with ≥ college level of education (4.74, 4.88, 4.84 versus 5.15; *p* ≤ .05) after adjustment for age. This significance, however, disappeared in the fully adjusted model.

**Table 5:** Geometric mean difference in adiponectin level for each socioeconomic status category compared to highest category based on general linear model

Adiponectin	Men				Women			
	Model:				Model:			
Income	^a^1	^b^2	^c^3	^d^4	^a^1	^b^2	^c^3	^d^4
≤ $19,999	3.91	3.78	4.17	3.88	4.98	5.33	5.33	5.11
$20,000–$49,999	3.25	3.24	3.70	3.46	4.94	5.53	5.53	5.17
≥$50,000 (referent)	3.02	3.07	3.59	3.31	4.88	5.48	5.48	5.17
*P* for linear trend	<.0001	<.0001	.001	.0008	.53	.20	.38	.7370
Education	^a^1	^b^2	^c^3	^d^4	^a^1	^b^2	^c^3	^d^4
<high school	3.98	3.62	4.07	3.71	5.18	4.74	5.28	4.94
High school or GED	3.26	3.28	3.71	3.53	4.97	4.88	5.42	5.04
Some college or vocational	3.07	3.21	3.73	3.44	4.64	4.84	5.39	5.26
≥College graduate (referent)	3.10	3.14	3.68	3.43	5.06	5.15	5.57	5.21
*P* for linear trend	<.0001	.006	.08	.14	.22	.05	.23	.12

*Abbreviations*: *GED* graduate equivalency diploma

^a^Model 1, unadjusted

^b^Model 2, adjusted for age

^c^Model 3, adjusted for age and health behavior (overweight, smoking status, alcohol consumption status, physical activity)

^d^Model 4, adjusted for age, health behavior and health status (HDL, LDL, triglycerides, hypertension status, type 2 diabetes status, HOMA-IR, CRP)

## Discussion

Consistent observations of SES gradients in health outcomes has led to the search for pathways through which social status may impact health-including biological mechanisms through which social characteristics and experiences may affect functioning and disease outcomes. One biological pathway that is gaining increasing interest is subclinical inflammatory processes that are thought to be involved in both the development and progression of a number of diseases, including cardiovascular, for which there are known SES gradients [5–10]. Adiponectin is an anti-inflammatory biomarker linked to CVD [12]. A low concentration of circulating adiponectin has been associated with a higher risk of CVD and its related risk factors, including hypertension, type 2 diabetes and obesity [12, 13, 26]. In addition, these outcomes are strongly patterned by SES [4]. African Americans have lower levels of adiponectin, higher prevalence of hypertension, type 2 diabetes, obesity, and lower SES is disproportionately prevalent in this population when compared to other racial/ethnic groups [15, 16]. The purpose of our study was to investigate the relationship of SES, based on annual household income and level of education, on level of adiponectin among African American men and women. Unlike the association between SES and other novel biomarkers [5–10], our findings revealed lower SES African American men had higher protective levels of adiponectin when compared to higher SES men. For example, African American men with the lowest household income had higher levels of adiponectin than those with the highest household income. Adjusting for health behavior and health status attenuated the association, but remained a strong statistical significance. The relationship regarding adiponectin and level of education was only significant in the crude and age adjusted models resulting in a mediating effect with health behavior and health status. There was no association between annual household income and differential levels of adiponectin among women. However, consistent with other studies assessing SES and biomarkers [5–9], women in our study with lower levels of education also had lower adiponectin levels compared to women with ≥ college level of education. However, this relationship disappeared after adjustments for health behavior and health status.

To our knowledge, there are only three publications that investigated the relationship between SES and adiponectin. Khanolkar, Vagero and Koupil investigated the association of occupational class and educational level among a sample of Swedish men 50 to 70 years of age [10]. They found no association between these SES measures and adiponectin levels. Researchers in South Africa investigated the association of adipokine levels and educational level between African and Asian-Indian women [18]. They also report no differential association between educational level and this adipokine. Buchan et al. report on the relationship of SES and adiponectin among a sample of adolescent boys and girls in Scotland [17]. Their findings revealed boys and girls from a lower SES had significantly lower adiponectin than those from a higher SES. This finding is consistent with our observation concerning women. Our findings concerning men, on the other hand, is contrary with the findings of Buchan et al. and other investigators assessing the relationship between SES and other novel biomarkers [5–10, 27]. These investigations revealed deleterious biomarker levels among the lower SES group. This was particularly evident regarding the association of annual household income. Our findings concerning the association of income and level of education among men may be due to how SES indices differentially affect health through inflammatory and anti-inflammatory biomarkers. Several studies have found that income and education are independently associated with health [28, 29], only one study has examined their shared or independent associations with a biomarker [30]. Results showed lower education predicted higher CRP levels independent of income in a sample of patients with heart disease [31]. However, racial differences were not reported. In African Americans, it may be that social position based on income among men affects health differently than educational status. Proximate factors associated with income may indeed be a stronger predictor of adiponectin than more distal factors related to education. Indeed, men in the lowest income category in our study had significantly higher protective levels of adiponectin than men in the highest income category after adjustments for health behavior and health status; BMI was also lower in this group but higher among men in the highest income category (Table 2). These findings suggest that higher income men may have an economic advantage and very little barriers to access and exposure to high fat diet unlike potential economic barriers experienced by men in the lowest income category.

### Strengthens and limitations

The strength of our investigation is that findings were from the largest community-based sample of African Americans, a cohort with strict protocol and high quality-control. It also addresses a risk factor (i.e. adiponectin) associated with CVD that disproportionately affects African Americans. In addition, it presents contrary new insight into historical findings concerning SES gradients and health-particularly among men. Finally, it is among the first to report on the association between SES and adiponectin based on a sample population in the United States. One limitation of the study is that findings cannot be generalizable to other ethnic groups. Secondly, this is a cross-sectional analysis; thus, we cannot establish a causal relationship between SES and adiponectin. Furthermore, residual confounders may have impacted the results. It is also important to mention that we use level of education and annual household income as proxy measures of SES and not a composite measure of SES. Although we adjusted for several known confounders, our study did not adjust for other factors such as dietary intake, sex hormones, and specific adiposity measures such as visceral fat. Finally, our study used total adiponectin rather than high molecular weight (HMW) adiponectin, which is considered the most biologically active form. This could potentially affect our findings since some studies suggest differences in biological activity between different isoforms of adiponectin and metabolic abnormalities [22]. However, findings also demonstrate that HMW does not provide more significant information than total adiponectin [32].

## Conclusion

The major finding of our study revealed that, after adjustments for age, health behavior and health status, adiponectin was significantly higher in African American men in the lowest category of income compared to those in the highest income category. Adiponectin was also higher among men with the lowest level of education after adjustment for age. Women with < high school and some college or vocational level of education had lower adiponectin than women with the highest level of education after age adjustment. Findings suggest a potential inverse biological pathway associated with annual household income as a measure of SES and adiponectin in men. Findings further suggest that perhaps intervention should be targeted to higher SES African American men to improve adiponectin level.

### Abbreviations

BMI, body mass index; CRP, C-reactive protein; CVD, cardiovascular disease; GED, graduate equivalency diploma; HDL, high-density lipoprotein; HOMA-IR, homoeostasis model assessment – insulin resistance; JHS, Jackson Heart Study; LDL, low-density lipoprotein; SES, socioeconomic status
